# Supplementary material for: Adaptive Laboratory Evolution and Carbon/Nitrogen Imbalance Promote High-Yield Ammonia Release in Saccharomyces cerevisiae
Source: Microorganisms. 2025 Jan 25;13(2):268. doi: 10.3390/microorganisms13020268 (PMC11858359; doi:10.3390/microorganisms13020268)

Supplementary material

Table S1. Conversion factor between OD660nm and dry weight for the *Saccharomyces cerevisiae* strains. The relationships were used to determine the DW (g/L) in culture. The linear regressions were performed with at least three data points.

Strain	gDW/OD	R^2
CBS 8066	0,198	0,999
CBS 8272	0,225	0,999
CBS 8267	0,216	0,997
BY 4742	0,204	0,996
T23D	0,210	0,988
CEN.PK 113-7D	0,196	0,989

Table S2. Detailed YP/YPD composition was expressed in g/L. * YPD differs from YP for the addition of 20 g/L glucose

Medium	Ammonia (g/L)	Trehalose (g/L)	Glycerol (g/L)	Acetic acid (g/L)	Amino acids (g/L)	Peptides (g/L)
**YP/YPD**	0,11 ± 0,02	0,98 ± 0,16	0,09 ± 0,01	0,11 ± 0,01	8,73 ± 0,14	12,72 ± 0,64

Table S3. Residual trehalose at the inoculation time and after 72 h of growth in YP buffered medium.

Strain	Trehalose (0 h)	Trehalose (72 h)
CBS 8066	0,86 ± 0,04	0,00
CBS 8272	0,84 ± 0,05	0,00
CBS 8267	0,89 ± 0,13	0,00
BY 4742	0,85 ± 0,08	0,00
T23D	0,9 ± 0,03	0,00
CEN.PK 113-7D	0,95 ± 0,04	0,00

Table S4. Residual amino acids after 72 h of growth in YP buffered medium.

Amino acids	YP Buffered	CBS 8066	CBS8272	CBS8267	BY4742	T23D	CEN.PK113-7D
Asp	0,226±0,032	0,194±0,022	0,216±0,021	0,167±0,024	0,155±0,026	0,304±0,01	0,262±0,06
Glu	1,051±0,108	0,544±0,086	0,989±0,04	0,712±0,004	0,702±0,034	0,945±0,024	0,898±0,078
Asn	0,257±0,019	0,193±0,093	0,236±0,016	0,245±0,036	0,193±0,027	0,266±0,015	0,228±0,007
Ser	0,443±0,035	0,347±0,022	0,382±0,02	0,339±0,024	0,292±0,003	0,351±0,016	0,343±0,012
His	0,22±0,002	0,121±0,006	0,103±0,033	0,159±0,081	0,158±0,051	0,171±0,014	0,171±0,02
Gly/Thr	0,683±0,004	0,788±0,089	0,683±0,051	0,737±0,04	0,603±0,01	0,696±0,035	0,689±0,043
Arg	0,901±0,06	0,859±0,024	0,865±0,067	0,959±0,005	0,788±0,044	0,881±0,027	0,919±0,03
Ala	0,846±0,05	0,92±0,042	0,869±0,145	1,014±0,093	0,798±0,076	0,973±0,026	0,97±0,007
Tyr	0,144±0,016	0,133±0,011	0,076±0,044	0,069±0,098	0,124±0,022	0,115±0,008	0,116±0,01
Val	0,503±0,002	0,612±0,017	0,536±0,039	0,611±0,054	0,5±0,049	0,539±0,014	0,529±0
Met	0,15±0,003	0,117±0,052	0,176±0,037	0,147±0,002	0,127±0,002	0,151±0,01	0,133±0,013
Trp	0,698±0,082	0,824±0,03	0,622±0,046	0,669±0,019	0,568±0,027	0,708±0,022	0,646±0,005
Phe	0,112±0,011	0,21±0,026	0,064±0,019	0,093±0,021	0,071±0,002	0,096±0,006	0,069±0,036
Ile	0,435±0,078	0,444±0,053	0,398±0,029	0,455±0,006	0,374±0,019	0,434±0,014	0,408±0
Leu	1,155±0,083	1,071±0,022	1,051±0,08	1,145±0,077	0,969±0,052	0,928±0,228	1,036±0,077
Lys	0,67±0,017	0,654±0,036	0,679±0,038	0,754±0,049	0,636±0,026	0,683±0,018	0,699±0,028
Pro	0,23±0,004	0,229±0,02	0,429±0,293	0,159±0,092	0,199±0,051	0,229±0,01	0,269±0,077

Table S5. Ammonia generated by CEN.PK113-7D and Evolved Strain in YPD medium at the end of growth (72 hours).

Strain	NH3 (g/L)
CEN.PK113-7D	0,04 ± 0,01
AAV1	0,04 ± 0,00
AAV2	0,05 ± 0,02
AAV3	0,05 ± 0,01
AAV4	0,05 ± 0,02
AAV5	0,07 ± 0,01
AAV6	0,05 ± 0,01
AAV7	0,07 ± 0,01

Figure S1. OD660nm vs dry cell weight for CEN.PK113-7D and CEN.PK113-7D-derived clones. The linear relationships were used to determine the DW (g/L) for flasks and fermentations.

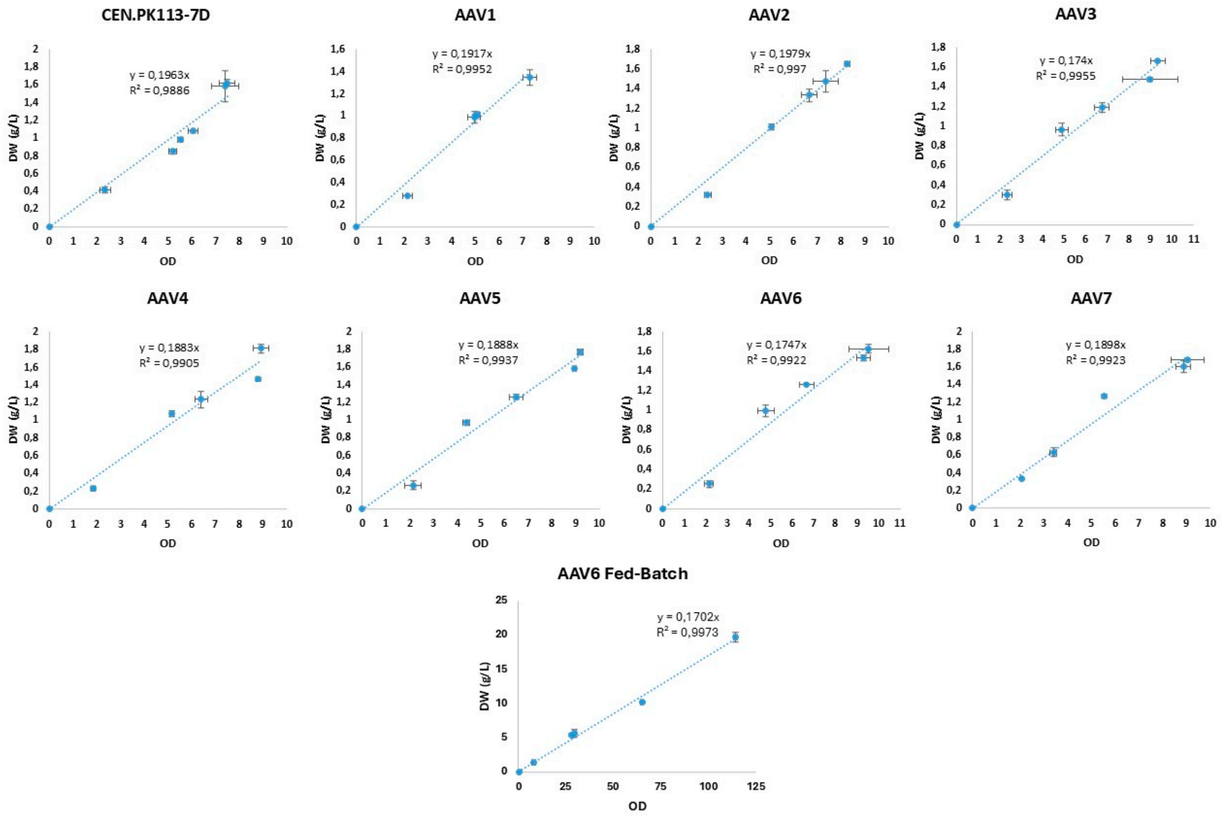

Figure S2. The quantification of free amino acid levels in YP medium after 72 hours of cultivation was measured using a high-performance liquid chromatography (HPLC) technique. Remarkably, the evolved strain exhibited a significantly higher uptake of the analyzed amino acids, such as aspartate, glutamate, asparagine, serine, arginine, alanine, and tryptophane.

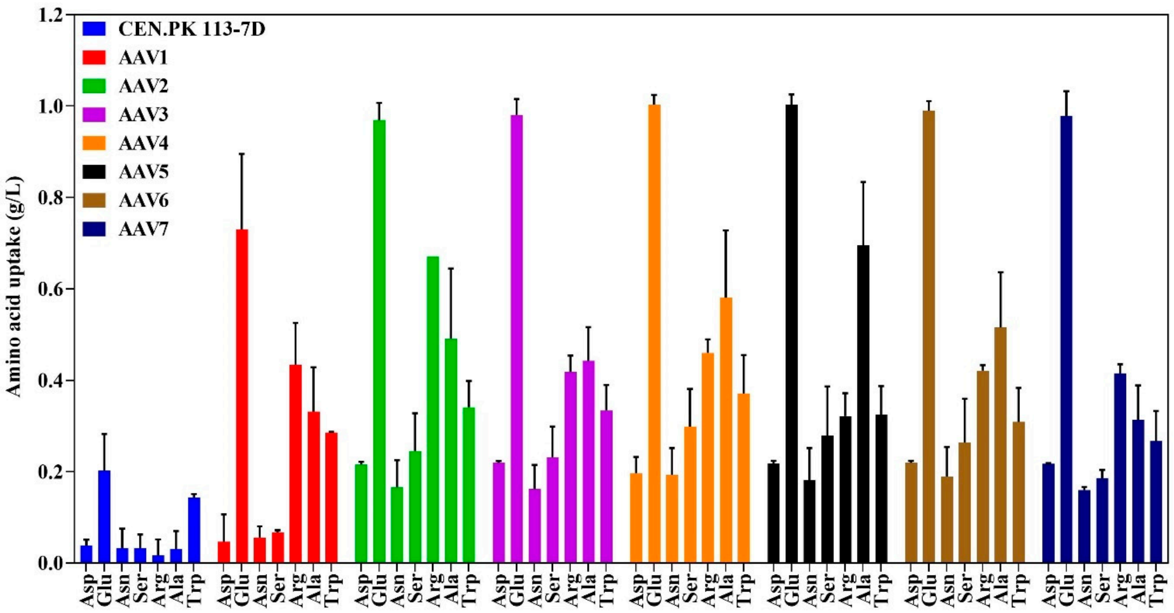

Figure S3. Quantification of free amino acid levels in a bioreactor (AAV6). A decrease in amino acid concentration was highlighted (aspartic acid, glutamic acid, asparagine, serine, arginine, alanine, and tryptophan). The asterisk (*) indicates significant differences ($p < 0.05$) using Student's t-test.

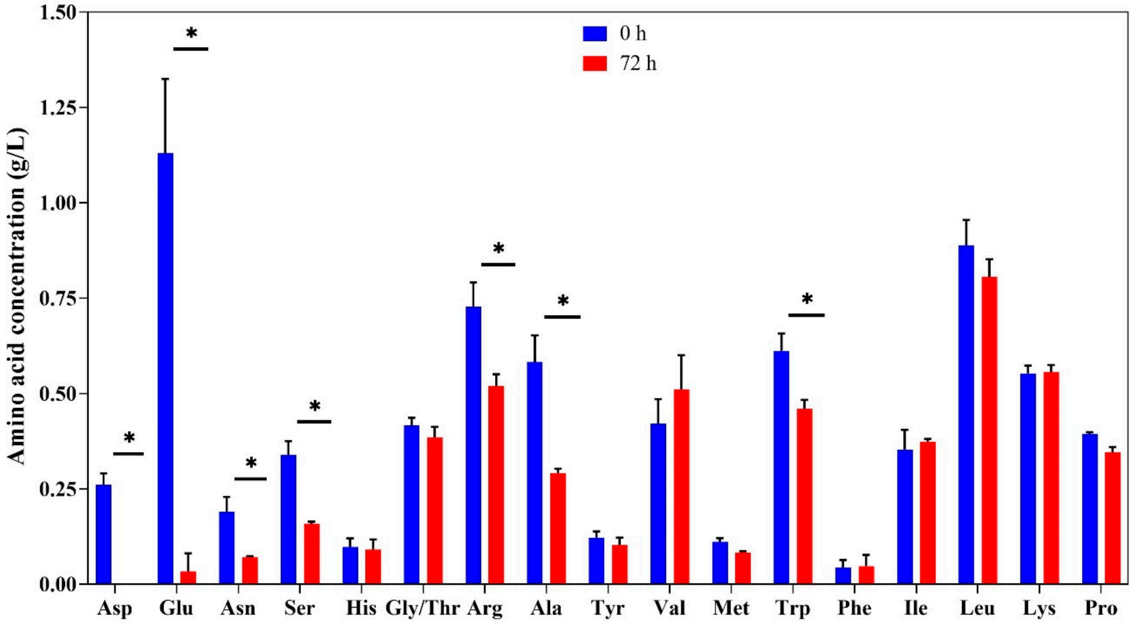

Figure S4. 3-step fermentation strategy with AAV6. Quantification of free amino acid levels before the addition of trehalose (ii) and at the end of growth (iii) in bioreactor. A pattern already observed during the batch cultivation was fully repeated. Glu and Ser were completely depleted, and a decrease in asp and ser was noticed. The asterisk (*) indicates significant differences ($p < 0.05$) using Student's t test.

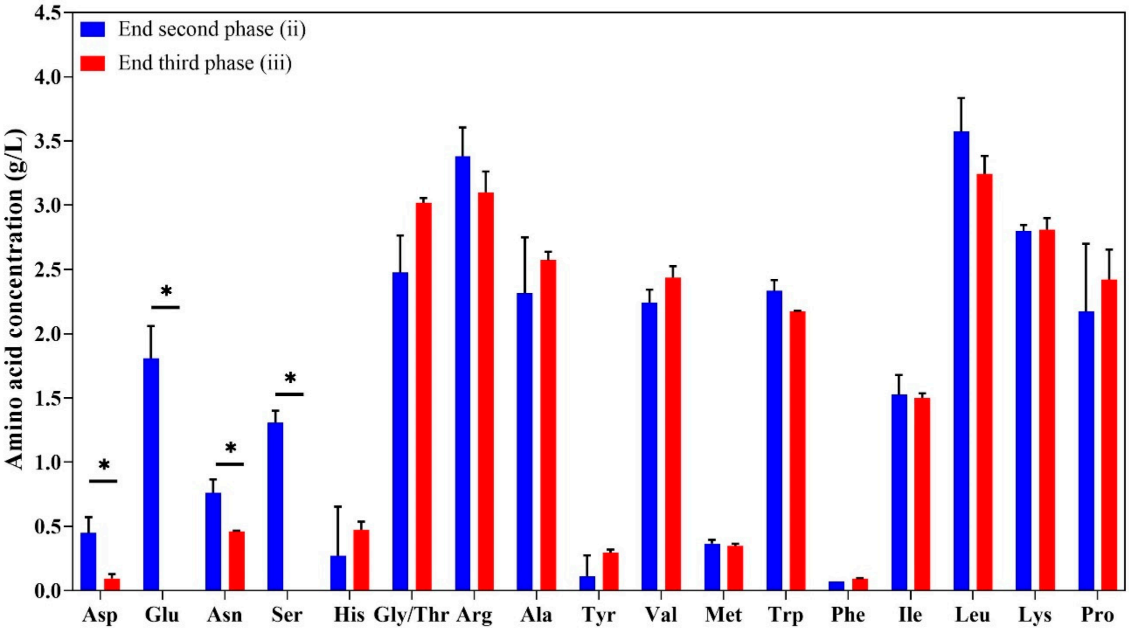

Supplement: Supplementary file 1 [file microorganisms-13-00268-s001.zip › microorganisms-3407781-supplementary.pdf]
